# Polyoxazoline-conjugated porcine serum albumin as an artificial plasma expander for dogs

**DOI:** 10.1038/s41598-023-35999-4

**Published:** 2023-06-14

**Authors:** Wataru Okamoto, Tomone Usui, Mai Hasegawa, Tatsuhiro Kobayashi, Junya Fujisawa, Kazuaki Taguchi, Kazuaki Matsumoto, Mitsutomo Kohno, Masayuki Iwazaki, Shotaro Shimano, Itsuma Nagao, Hiroto Toyoda, Naoki Matsumura, Hirotaka Tomiyasu, Ryota Tochinai, Teruyuki Komatsu

**Affiliations:** 1 Department of Applied Chemistry, Faculty of Science and Engineering, Chuo University, 1-13-27 Kasuga, Bunkyo-ku, Tokyo, 112-8551 Japan; 2 Division of Pharmacodynamics, Faculty of Pharmacy, Keio University, 1-5-30 Shibakoen, Minato-ku, Tokyo, 105-8512 Japan; 3 Department of General Thoracic Surgery, Saitama Medical Center, Saitama Medical University, 1981 Kamoda, Kawagoe-shi, Saitama 350-8550 Japan; 4 Department of Thoracic Surgery, School of Medicine, Tokai University, 143 Shimokasuya, Isehara-shi, Kanagawa 259-1193 Japan; 5 Department of Veterinary Internal Medicine, Graduate School of Agriculture and Life Sciences, The University of Tokyo, 1-1-1 Yayoi, Bunkyo-ku, Tokyo, 113-8657 Japan; 6 Department of Veterinary Pathophysiology and Animal Health, Graduate School of Agriculture and Life Sciences, The University of Tokyo, 1-1-1 Yayoi, Bunkyo-ku, Tokyo, 113-8657 Japan

**Keywords:** Animal biotechnology, Biomaterials, Biomimetics

## Abstract

Veterinary medicine has made tremendous progress for domestic dogs, which are irreplaceable family members enriching human life. Nevertheless, no adequate supply system exists for their blood products. This study examined the synthesis, structure, safety, and efficacy of poly(2-ethyl-2-oxazoline)-conjugated porcine serum albumin (POx-PSA) as an artificial plasma expander for dogs. The aqueous POx-PSA solution showed moderately high colloid osmotic pressure and good blood cell compatibility. Actually, lyophilized powder stored for 1 year can regenerate into a homogeneous solution. The circulation half-life of POx-PSA in rats was 2.1-fold longer than that of naked PSA. Rats produced neither anti-PSA IgG antibody nor anti-POx IgG antibody, which suggests excellent immunological stealth properties of POx-PSA. Complete resuscitation of hemorrhagic shock in rats was achieved soon after injection of POx-PSA solution. Serum biochemistry tests and histopathological observations indicated no abnormality in the related organs. When POx-PSA was administered to dogs intravenously, (i) no serum biochemical or hematological alteration was observed, also (ii) no overt deterioration of animal health was observed. These results indicate that POx-PSA has potential as an artificial plasma expander for dogs.

## Introduction

In modern society, domestic cats and dogs are important family members that enrich human life. Advances in veterinary medicine, even in areas of preventive medicine and health consciousness, have extended pet lifespans. As one might expect, cases of illness are becoming increasingly complicated. Demand for blood transfusion treatment continues to grow. Nevertheless, no sufficient system exists for supplying blood products such as serum albumin prepared from animal blood. The purpose of administering albumin is to maintain the colloid osmotic pressure (COP) and to secure the circulating blood volume (circulating plasma volume). Specifically, albumin preparations should be administered when hypoalbuminemia occurs because of bleeding, increased capillary permeability, decreased albumin synthesis in the liver, excess loss from the kidneys and intestines, and accelerated metabolism. For actual veterinary practice, hydroxyethyl starch (HES) and human serum albumin (HSA) have often been forced for use as plasma substitutes. Several HES products with different molecular weights can be purchased on the market. Voluven [6% HES in saline (0.9% NaCl) solution] is a typical formulation. This nonionic starch derivative, however, causes side effects such as renal dysfunction, blood coagulation disorders, shock, and elevated amylase^1–7^. Multiple meta-analyses in fields of human medicine have shown high blood bleeding in HES-treated groups^1,4,8,9^. Administration of a poorly metabolized HES 200 reduced factor VIII and von Willebrand factor by 80%, provoking inhibition of the endogenous coagulation cascade^10,11^. Furthermore, HES engenders morphological change of glycoprotein IIb/IIIa and thereby retards platelet aggregation. Infusion of HSA in cats and dogs definitely increases blood albumin concentration and circulating blood volume. It is also effective for recovery from hypoalbuminemia. Nonetheless, an acute or delayed side reaction occurred in 20–30% of cases^12–14^. As a heterologous protein, HSA elicits an immune response to a greater or lesser extent. Cohn et al. reported that anti-HSA antibodies were produced 14 days after infusion to healthy dogs^15^. Bovine serum albumin (BSA) from cattle and porcine serum albumin (PSA) from swine are readily available, although their immunogenicity remains a difficulty that must be overcome.

**Figure 1:**
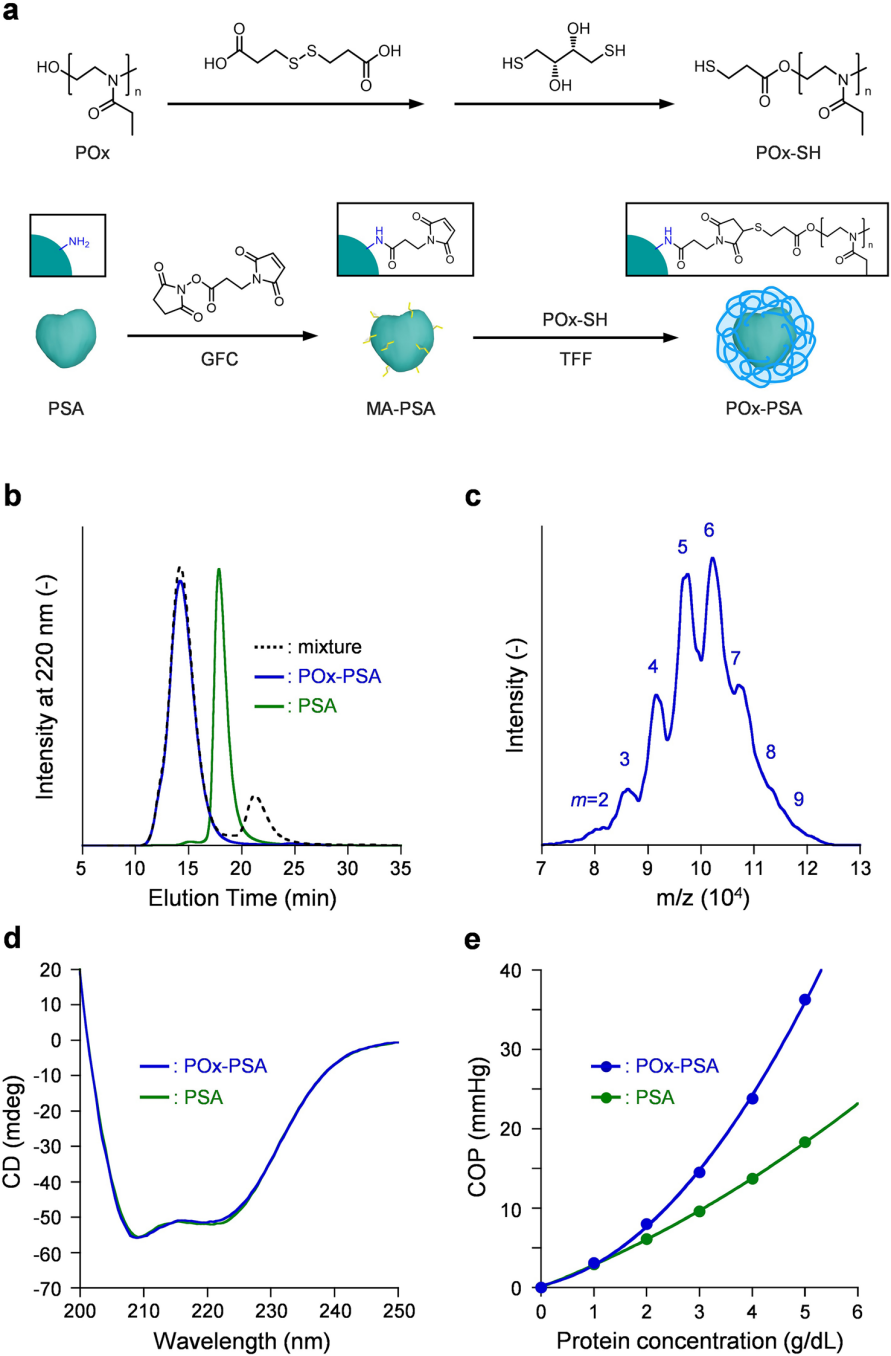
Synthesis and physicochemical properties of POx-PSA. (**a**) Synthesis scheme of POx-PSA using a cross-linking agent SMP. (**b**) SEC profiles of the reaction mixture and purified POx-PSA at 25 °C. (**c**) MALDI-TOFMS of POx-PSA. (**d**) CD spectra of POx-PSA and PSA ([PSA] = 4 μM, in PBS, pH 7.4, at 25 °C). (**e**) COP of POx-PSA and PSA as a function of the protein concentration (in PBS, pH 7.4, at 25 °C).

Surface modification of therapeutic proteins with poly(ethylene glycol) (PEG), a widely exploited biocompatible polymer, improves many shortcomings such as rapid clearance, proteolysis, and immunogenicity^16^. After producing a camouflaging shield around the protein, PEG increases the hydrodynamic size and inhibits renal glomerular filtration. Moreover, this polymer layer masks the protein antigens, therefore providing immunological stealth property. Although PEG-conjugated BSA or PSA might seem to be a good candidate as an artificial plasma expander for dogs, several researchers have reported that anti-PEG antibodies (IgM and IgG) are formed in the body^17,18^. Furthermore, repeated infusion of the PEGylated protein engenders kidney cell vacuolization^19^. Given that background, great interest has arisen recently in development of an artificial plasma expander for pet animals with sufficient safety. Poly(2-ethyl-2-oxazoline) (POx), a potential alternative to PEG^20^, can be synthesized reliably by living cationic ring-opening polymerization of 2-ethyl-2-oxazolines^21^. Its physicochemical features such as its nonionic character, high solubility in various solvents, and main chain flexibility are comparable to those of PEG^22^. In addition, POx is characterized by its low viscosity, high stability at room temperature, ready degradation in the body, and lack of peroxide formation. POx-conjugated proteins, enzymes, and peptides show a long blood circulation time and sufficient safety while maintaining their original therapeutic effects^20,23^. The immunological stealth capability of POx is regarded as superior to that of PEG^20,24^. In view of those earlier studies’ results, we decided to employ a combination of PSA from specific pathogen free (SPF) swine and POx. This report describes the synthesis, structure, safety, and efficacy of POx-conjugated PSA (POx-PSA, Fig. 1a) as an artificial plasma expander for dogs.

## Results

### Synthesis of POx-PSA

Unique POx bearing a sulfhydryl group terminus, POx-SH, was synthesized by condensation of POx with 3,3′-dithiodipropionic acid (DTDPA) followed by cleavage of the central disulfide bond (80% yield) (Fig. 1a). The sulfhydryl ratio of POx-SH was 100%. As core albumin, we used special PSA obtained from porcine blood of SPF swine, which had been reared in clean piggeries and which were guaranteed to be free of certain diseases such as mycoplasma pneumonia. We purified PSA from the porcine serum using a simple heating procedure. The porcine plasma solution with an excess amount of sodium caprylate was heated at 70 °C to denature other proteins. After discarding the precipitate, the supernatant was subjected to anion exchange chromatography (AEC). The isolated PSA was analyzed using SDS-PAGE and size exclusion chromatography (SEC) on an HPLC system to confirm the protein integrity (purity 99%).

Maleimido-activated PSA (MA-PSA) prepared using a cross-linking agent, *N*-succinimidyl 3-maleimidopropionate (SMP), was reacted with POx-SH (Fig. 1a). Formation of POx-PSA was confirmed by SEC measurement. The dominant peak appeared in a higher-molecular-weight region compared to that of PSA, indicating several POx chains bindings to PSA (Fig. 1b). Purification of POx-PSA was achieved using tangential flow filtration (TFF). No peak of unreacted POx-SH was observed on the SEC curve of the purified POx-PSA (> 99% yield).

### Structure and physicochemical properties

Dynamic light scattering (DLS) measurement showed that the average particle size of POx-PSA was 13.5 nm, which is markedly larger than that of naked PSA (7.8 nm). From gravimetric analysis of the freeze-dried powder, the average binding number of POx (*m*) was calculated as 5.9 ± 0.4. When using matrix-assisted desorption ionization time-of-flight mass spectroscopy (MALDI-TOFMS), multiple peaks based on POx-PSA with different polymer numbers appeared exactly every 5300 Da (Fig. 1c). The highest peak at 102,710 Da corresponds to the six POx conjugate. The peak pattern simulation and curve fitting enables us to ascertain the *m* number as 5.7. This value is well consistent with the gravimetric analysis results explained above. The zeta potential of POx-PSA was 0.5 mV. The negatively charged surface of PSA (− 20.3 mV) appears to be well covered by the nonionic neutral POx. The CD spectrum of POx-PSA exhibited a strong signal with double local minima at 208 and 222 nm, which was identical to that of PSA (Fig. 1d). The secondary structure of the protein core was retained even after polymer wrapping.

The COP of the POx-PSA solution was shown as a function of PSA concentration (Fig. 1e). The line deviated upward from the linear correlation of ideal solution behavior because of the large second virial coefficient. The POx-PSA solution ([PSA] = 5 g/dL) showed COP of 36 mmHg, which was 2.0-fold greater than that of PSA (5 g/dL, 18 mmHg). Lyophilization of aqueous POx-PSA solution under reduced pressure yielded white powder, which can be stored for 1 year at 4 °C. Addition of PBS solution to the powder regenerated the homogeneous clear solution. The SEC and CD patterns of the redispersed POx-PSA were identical to those found before freeze-drying.

### Hemocompatibility and blood coagulation

**Figure 2:**
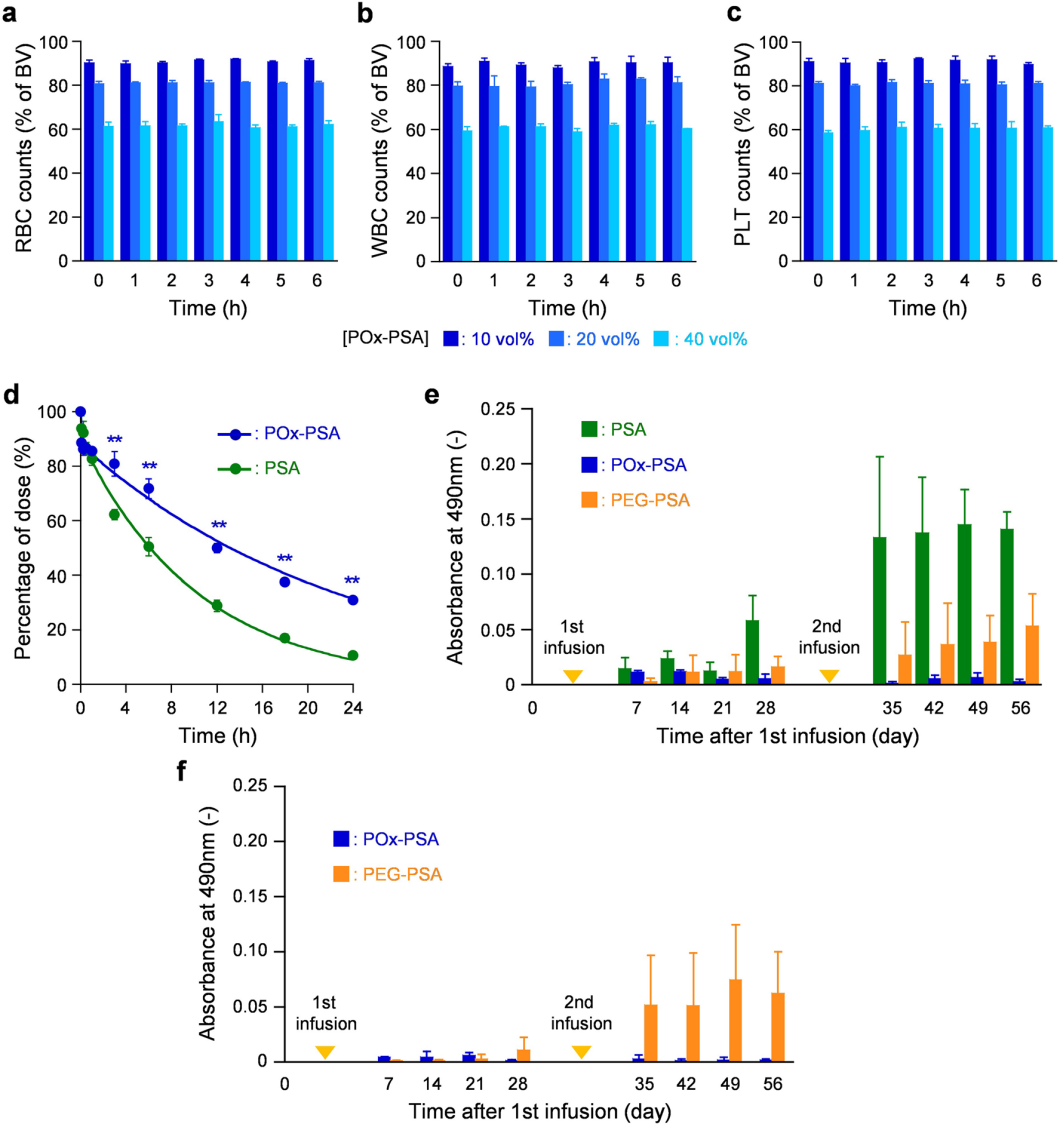
Hemocompatibility, circulation persistence, and immunogenicity of POx-PSA. (**a**) RBC, (**b**) WBC, and (**c**) platelet counts of rat blood samples after mixing with POx-PSA solution ([POx-PSA] = 10, 20, and 40 vol%). Basal values (BV) were 6.1 × 10^6^ cells/μL in the RBC group, 3.8 × 10^3^ cells/μL in the WBC group and 8.0 × 10^5^ cells/μL in the platelet group. Each datum represents mean ± SD (*n* = 3). (**d**) Relative plasma concentration of POx-PSA(Cy5.5) after intravenous administration to rats. Each point represents the mean ± SD (*n* = 4). (**e**) Relative immunogenicity of POx-PSA, PEG-PSA, and PSA in rats. Absorbance at 490 nm corresponds to the amount of anti-PSA IgG antibody in the collected blood sample. Each datum represents mean ± SD (*n* = 3). (**f**) Relative immunogenicity of POx-PSA and PEG-PSA in rats. Absorbance at 490 nm corresponds to the amount of anti-POx IgG or anti-PEG IgG antibody in the collected blood sample. Each datum represents mean ± SD (*n* = 3).

To assess the hemocompatibility of POx-PSA, we measured the blood cell counts in a mixture suspension of rat blood and POx-PSA (blood/POx-PSA = 9/1, 8/2, and 6/4, v/v) in vitro. The RBC, white blood cell (WBC), and platelet (PLT) counts decreased in proportion to their respective dilution to approximately 90, 80, and 60% of the basal values, and remained constant for 6 h at 37 °C (Fig. 2a–c).

The influence of POx-PSA on blood coagulation was investigated using measurements of the prothrombin time (PT) and the activated partial thromboplastic time (APTT). The PT tests assess the extrinsic and common pathways of the coagulation cascade. The APTT tests evaluates the intrinsic and common pathways. The PT and APTT of rat blood/POx-PSA mixtures (9/1, 8/2, and 6/4, v/v) were slightly higher than the basal values, depending on the POx-PSA volume ratio (Fig. S1). By contrast, the APTT values were not increased by the dilution with HES.

### Circulation persistence

The Cy5.5-labeled fluorescent POx-PSA [POx-PSA(Cy5.5)] was injected into rats to assess blood retention. Fluorescence intensity of the plasma collected from the rats (λ_em_ = 710 nm) decreased gradually over time (Fig. 2d). The time course of the POx-PSA concentration exhibited a biphasic profile composed of a distribution phase (α phase) and an elimination phase (β phase). The pharmacokinetic parameters were ascertained using a two-compartment model. The t_1/2_ of the β phase (15 h) was found to be 2.1-fold longer than that of naked PSA (t_1/2_ = 7 h) (Table 1). The POx-PSA group showed higher area-under concentration–time curve (AUC) value and mean residence time (MRT) (blood retention indexes), and lower clearance (CL_tot_) and volume of distribution at steady state (V_dss_) (drug metabolism rate indexes) compared to the values found for the PSA group.

**Table 1:** Pharmacokinetic parameters of POx-PSA after intravenous administration to rats (*n* = 4).

Formulation	t_1/2_ (h)	MRT (h)	CL_tot_ (mL/h)	V_dss_ (mL)	AUC (h% of dose/mL)
PSA	7	11	1.0	9.1	100
POx-PSA	15	22	0.5	7.8	183

### Immunogenicity evaluation in rats

The camouflaging ability of POx to mask the immunogenicity of PSA was evaluated using IgG assays after injection of the POx-PSA solution into rats. The formulation was administered to rats two times: on days 0 and 28. Observations continued for 56 days. In the PSA group, the amount of anti-PSA IgG antibody increased from 7 days after the first administration; it then rose very quickly by the second administration (Fig. 2e). Because of the existence of anti-PSA IgG antibody at day 28, the second administration of PSA caused a severe immune reaction and provoked further antibody generation. In stark contrast, almost no anti-PSA IgG antibody was found in the POx-PSA group for 56 days, even after the second administration. Injection of PEG-conjugated PSA (PEG-PSA) produced anti-PSA IgG antibody. The degree of production was almost one-third of that observed for naked PSA at day 56. It is particular interesting that anti-POx IgG antibody was not observed after infusion of POx-PSA (Fig. 2f). PEG-POx infusion, however, engendered anti-PEG IgG antibody formation. No abnormality was found in the biochemical and pathological assessments of the POx-PSA group, PEG-PSA group, or PSA group 56 days after administration.

### Resuscitation from hemorrhagic shock rats

In the hemorrhagic shock rat model prepared by 50 vol% blood bleeding, all animals died within 20 min if not resuscitated. Administration of resuscitative fluids [POx-PSA or HES (Voluven)] significantly improved the survival rate. All rats of the POx-PSA and HES groups survived for 2 h, suggesting the importance of restoring blood volume. The initial data before the 50 vol% blood bleeding were defined as a basal value (baseline).

#### Circulation parameters

**Figure 3:**
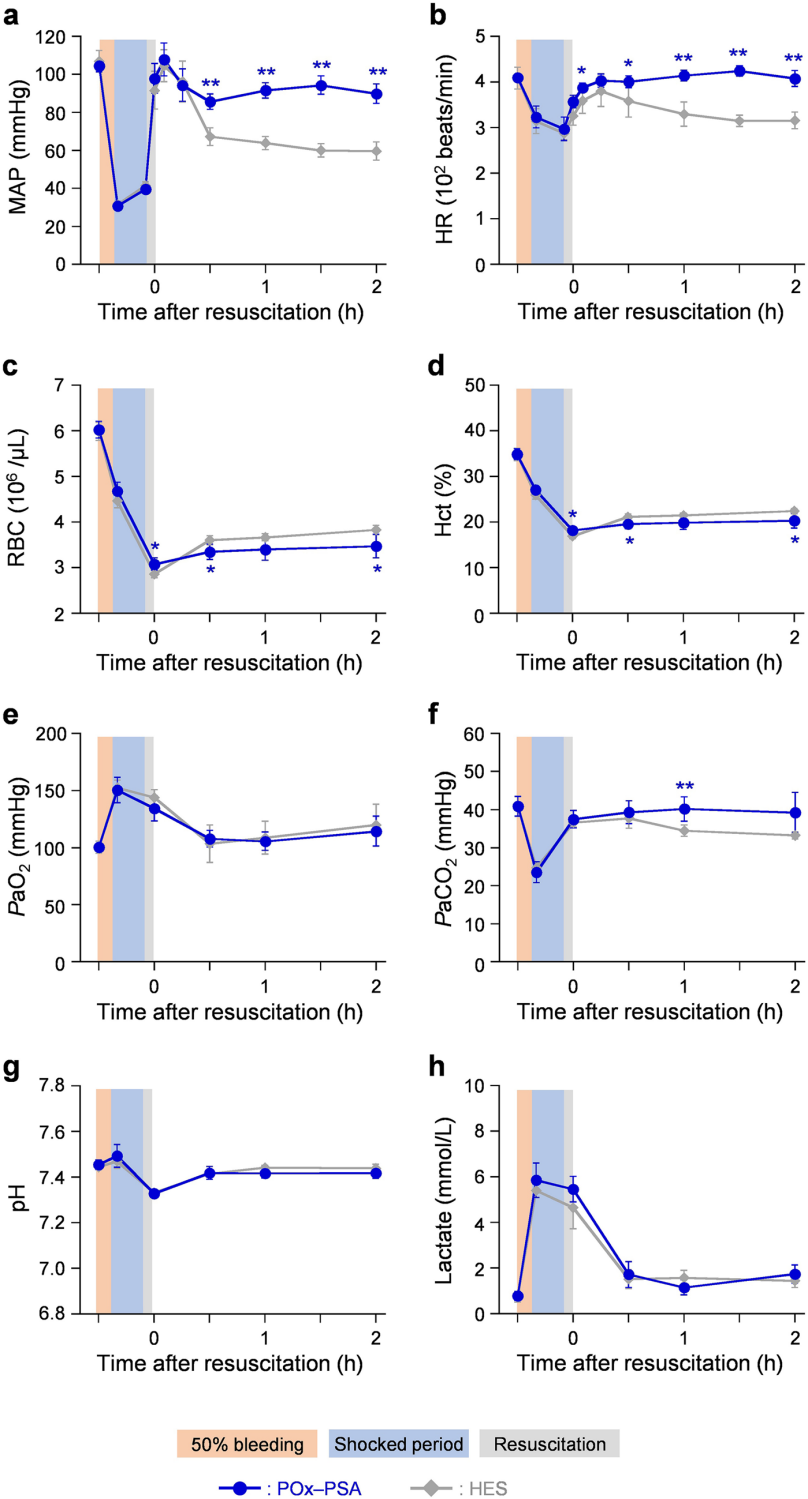
Changes of circulation parameters, hematology parameters, and blood gas parameters. Changes of (**a**) MAP, (**b**) HR, (**c**) RBC, (**d**) Hct, (**e**) *P*aO_2_, (**f**) *P*aCO_2_, (**g**) pH, and (**h**) lactate of anesthetized rats in hemorrhagic shock and after resuscitation. Each datum represents mean ± SD (*n* = 6). *P < 0.05, **P < 0.01 vs. HES group.

Mean arterial pressure (MAP) (106 ± 5 mmHg) decreased to 31 ± 1 mmHg after blood withdrawal (severe hemorrhagic shock) recovered immediately by injection of the POx-PSA solution and remained constant in the range of 86–107 mmHg until measurements were completed (Fig. 3a). In the HES group, MAP recovered soon after resuscitation, although it declined to 67 ± 5 mmHg after 30 min.

The decrease of the heart rate (HR) (319 ± 26 beats/min) because of blood loss recovered to 403 ± 15 beats/min 15 min after POx-PSA administration (Fig. 3b). The values remained high, at 401–424 beats/min, during the observation. In the HES group, the increased HR by the HES infusion (380 ± 34 beats/min) decreased slowly to 316 ± 19 beats/min after 2 h. Those values were considerably lower than the values of the POx-PSA group.

#### Hematological parameters

The RBC count and hematocrit (Hct) value declined after the formulation infusion to 51–52% of the baselines (Fig. 3c,d). This result means that 30 vol% administration (after 50 vol% blood withdrawal) was sufficient for recovery to the original volume of the circulating blood. In the POx-PSA group, these parameters were constant for 2 h, suggesting maintenance of the circulating blood volume. In the HES group, the RBC counts increased slightly after 1 h (Fig. 3c).

#### Blood gas parameters

The arterial blood O_2_ partial pressure (*P*aO_2_) (101 ± 4 mmHg) increased to 152 ± 9 mmHg after blood withdrawal (Fig. 3e). The arterial blood CO_2_ partial pressure (*P*aCO_2_) (41 ± 2 mmHg) decreased markedly to 24 ± 2 mmHg after blood bleeding (Fig. 3f). Increased *P*aO_2_ and decreased *P*aCO_2_ during hemorrhage are usually attributed to the relative hyperventilation caused by reduced cardiac output and subsequent loss of pulmonary blood flow. In the POx-PSA group and HES group, *P*aO_2_ returned respectively to 108 ± 7 and 104 ± 16 mmHg at 0.5 h after administration. Both values were nearly equal to those of the initial levels before hemorrhage. In the POx-PSA group, *P*aCO_2_ returned to 37 ± 2 mmHg soon after administration and remained steady during experimentation. In the HES group, however, the *P*aCO_2_ (37 ± 1 mmHg) recovered by the HES infusion decreased again to 33 ± 1 mmHg (after 2 h), which was lower than the value of the POx-PSA group.

The decreased pH after blood withdrawal and subsequent administration suggested the occurrence of mild acidosis in both the POx-PSA group and HES group (Fig. 3g). The values recovered respectively to 7.42 ± 0.03 and 7.44 ± 0.02 after 0.5 h from administration. Hypotension generally caused organ hypoperfusion, leading to a rise of the plasma lactate concentration. In fact, the lactate value (0.7 ± 0.2 mmol/L) was increased to 5.6 ± 0.6 mmol/L by blood withdrawal (Fig. 3h). In the POx-PSA group and the HES group, the values returned to the baseline by 0.5 h after injection and remained constant thereafter for 2 h.

#### Serum biochemistry tests

**Figure 4:**
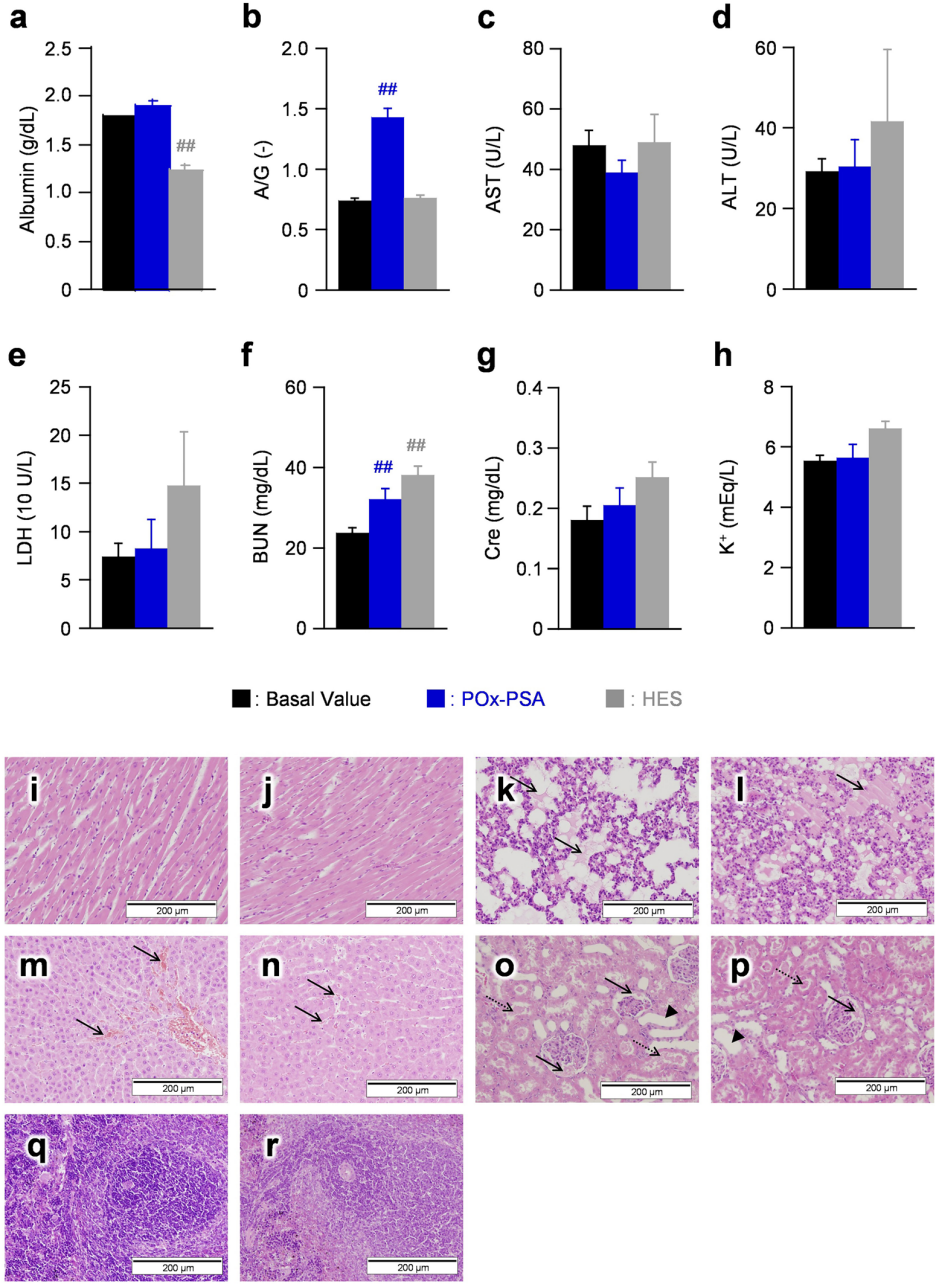
Serum biochemical tests and histopathological examinations. (**a**) albumin, (**b**) A/G ratio, (**c**) AST, (**d**) ALT, (**e**) LDH, (**f**) BUN, (**g**) Cre, and (**h**) K^+^ of anesthetized rats 2 h after administration. Each datum represents mean ± SD (*n* = 6). #*P* < 0.05, ##*P* < 0.01 vs. pre-hemorrhage group. Histopathological images of rats: (**i**,**j**) heart, (**k**,**l**) lung, (**m**,**n**) liver, (**o**,**p**) kidney, and (**q**,**r**) spleen [(**i**,**k**,**m**,**o**,**q**) HES group and (**j**,**l**,**n**,**p**,**r**) POx-PSA group] 2 h after administration. Edematous change (solid arrows in **k** and **l**) was found in the lung. Sinusoidal dilatation with congestion (solid arrows in **m** and **n**) was found in the liver. Bowman’s space dilatation, hyaline cast (solid arrows in **o** and **p**), hyaline cast in proximal tubules (dotted arrows in **o** and **p**), and dilatation of distal tubules (triangle in **o** and **p**) were found in the kidney.

Arterial blood samples withdrawn from the rats at 2 h after administration were subjected to serum biochemistry tests. The initial data before the 50 vol% blood bleeding were designated as a basal value (baseline). In the POx-PSA group, the albumin concentration (1.9 ± 0.1 g/dL) remained unaltered because the sample is rich in albumin (Fig. 4a). The albumin/globulin (A/G) ratio became almost double that of the baseline because of the 50% reduction of the globulin concentration (Fig. 4b). In the HES group, the albumin concentration was lower than the basal value. The A/G ratio was identical to the baseline value.

Aspartate transaminase (AST) is an enzyme existing in the liver, myocardium, skeletal muscle, and RBCs. Alanine transaminase (ALT), a liver enzyme, serves as a liver function index. Lactate dehydrogenase (LDH) is an enzyme present in the liver, kidneys, myocardium, skeletal muscle, and RBCs. They are released into the bloodstream when organs and tissues are damaged. The AST, ALT, and LDH values in the POx-PSA group were found to be almost identical to baseline values (Fig. 4c–e). In contrast, the HES group showed higher ALT (42 ± 18 U/L) and LDH (147 ± 56 U/L) than the initial values. Blood urea nitrogen (BUN), creatinine (Cre), and K^+^ are renal function indexes. The BUN value in the POx-PSA group (32 ± 3 mg/dL) was slightly higher than the baseline value, but lower than that found for the HES group (38 ± 2 mg/dL) (Fig. 4f). The BUN, Cre, and K^+^ values in the HES group were greater than the initial levels (Fig. 4f,g,h).

#### Histopathological examination

Organs removed from the rats after observation for 2 h were stained with hematoxylin/eosin and were examined microscopically. In neither the POx-PSA group nor HES group did the heart or spleen show any morphological abnormality (Fig. 4i,j,q,r). Edematous changes were observed in the lungs (Fig. 4k,l). Minor sinusoidal dilatation (with congestion) was found in the liver (Fig. 4m,n). In kidneys, Bowman’s space dilatation, hyaline casts, hyaline casts in proximal tubules, and dilatation of distal tubules were observed (Fig. 4o,p). However, we were unable to find any qualitative difference between the POx-PSA group and the HES group.

### Safety evaluation in healthy beagle dogs

As a safety evaluation, POx-PSA ([PSA] = 5 g/dL, in saline solution) was administered to three beagle dogs (top-loaded, 5 mL/kg, dogs D, E, and F). Their clinical symptoms and serum biochemistry were then observed for 28 days. PSA (5 g/dL, in saline solution) was also injected to the other three dogs as a control group (top-loaded, 5 mL/kg, dogs A, B, and C).

#### Clinical symptoms and medications

The dogs receiving the PSA solution (dogs A, B, and C) showed lethargy, anorexia, vomiting, diarrhea, and swelling of the superficial lymph nodes (Table 2). Furthermore, dogs A and C showed an enlarged spleen; dog B showed proteinuria, possibly because of thrombosis. All dogs that had shown severe allergic reactions were treated with a corticosteroid drug (prednisolone). Without treatment, worse allergic reactions might have occurred. The dogs in the PSA group were medicated with several administrations of antiemetic (maropitant), gastroprokinetic (metoclopramide), antiplatelet (clopidogrel), antibacterial (enrofloxacin), and angiotensin II receptor antagonist (telmisartan). In contrast, the clinical symptoms were very mild in dogs of the POx-PSA group (Table 2). Dog D had mild vomiting. Dog F, which had mild anorexia and vomiting, received one administration of antiemetic medication (maropitant). No other abnormality was found. No other therapeutic treatment was administered.

**Table 2:** Clinical symptoms and details of medication after POx-PSA or PSA administration to dogs (*n* = 3).

Formulation	Dog	Clinical symptoms [time after administration (day)]	Medication details [time after administration (day)]
PSA	A	Lethargy and anorexia [10–18] Vomiting and diarrhea [10–20] Swellings of superficial lymph nodes [10–21] Enlarged spleen [10–21]	Maropitant [10–16] Metoclopramide [15–16] Prednisolone [18–24]
	B	Lethargy and anorexia [10–18] Vomiting and diarrhea [10–19] Swelling of superficial lymph nodes [10–15] Enlarged spleen [10–15] Suspected thrombosis [14–15] Proteinuria [17–28]	Maropitant [11–16] Clopidogrel [14–26] Enrofloxacin [15–16] Telmisartan [17–28] Prednisolone [15–30]
	C	Lethargy and anorexia [17–20] Vomiting and diarrhea [17–20] Swelling of superficial lymph nodes [17–19]	Maropitant [19] Prednisolone [17–21]
POx-PSA	D	Mild vomiting [4]	‒
	E	‒	‒
	F	Mild anorexia [10–11] Mild vomiting [10–11]	Maropitant [11]

#### Hematological parameters and albumin concentration

**Figure 5:**
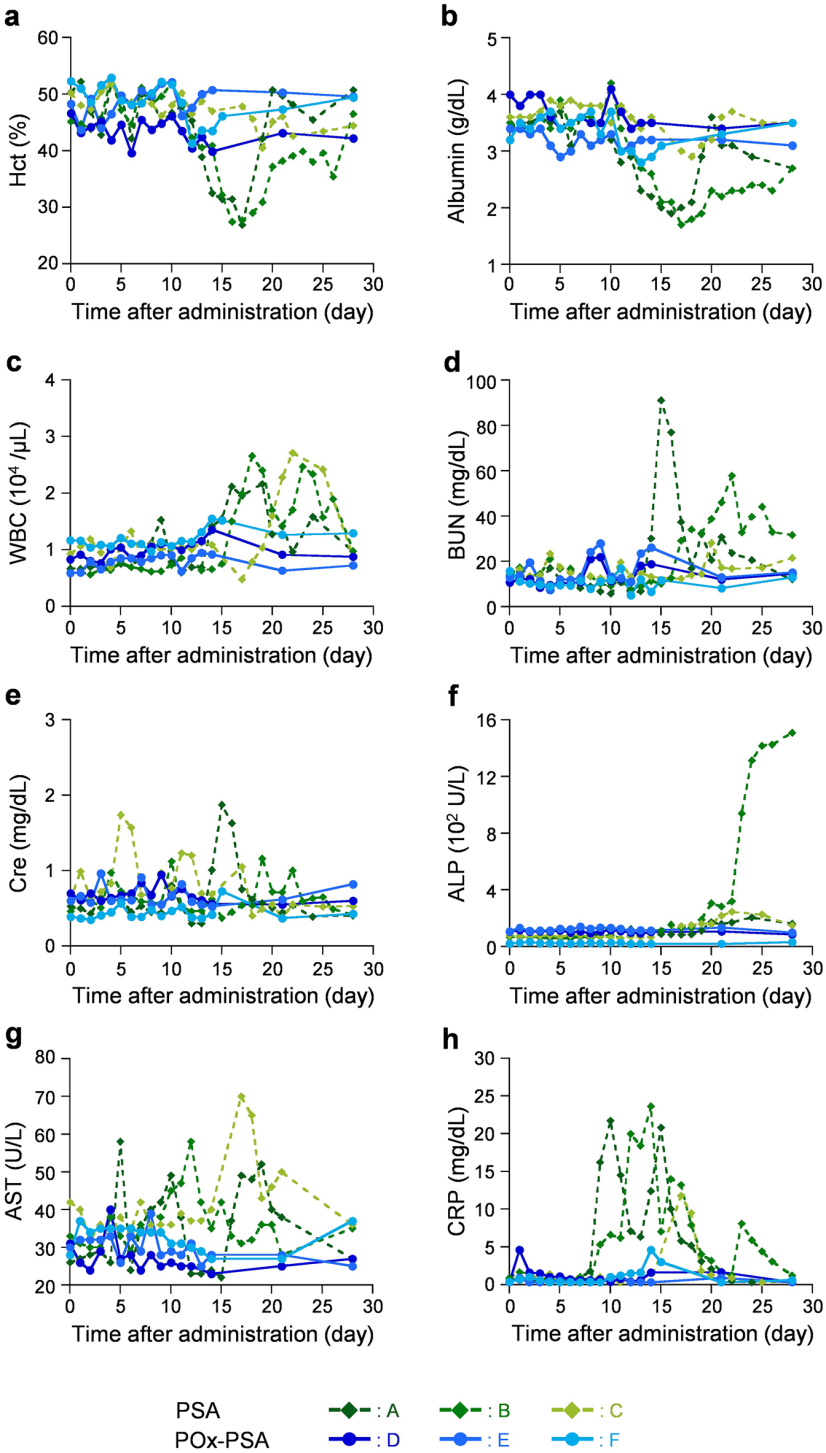
Changes of hematology parameters and serum biochemistry data. Changes of (**a**) Hct, (**b**) albumin, (**c**) WBC, (**d**) BUN, (**e**) Cre, (**f**) ALP, (**g**) AST, and (**h**) CRP of dogs after intravenous administration with POx-PSA or PSA solution.

The Hct and albumin concentrations of two dogs in the PSA group (dogs A and B) decreased respectively to 27% and 1.7–1.9 g/dL at 16–17 days after administration (Fig. 5a,b). The WBC counts in dogs A, B, and C had increased to 2.2, 2.7, and 2.7 × 10^4^/μL, respectively, 19, 18, and 22 days after administration (Fig. 5c). Results suggest that the PSA infusion caused inflammation, anemia, and hypoalbuminemia. On the one hand, all dogs (dogs D, E, and F) in the POx-PSA group kept these values within the standard ranges (Hct, 37–55%; albumin, 2.6–4.0 g/dL; WBC, 0.6–1.7 × 10^4^/μL).

#### Renal and hepatic parameters

The BUN values of dogs A and B in the PSA group increased to 91 and 58 mg/dL, respectively, 15 and 22 days after administration (Fig. 5d). The Cre values also rose to 1.9 and 1.7 mg/dL, respectively, 15 and 5 days after administration (Fig. 5e). In contrast, the POx-PSA group (dogs D, E, and F) were found to have the parameters within the reference values (BUN, 9–29 mg/dL; Cre, 0.4–1.4 mg/dL). The POx-PSA infusion did not affect kidney functions.

The ALP values, a liver enzyme, of dogs A, B, and C increased to 200 U/L 20 days after administration in the PSA group (Fig. 5f). Dog B showed dramatic elevation to 1400–1500 U/L, which was probably caused by treatment with corticosteroids. The AST values of dogs A, B, and C rose to 52, 58, and 70 U/L, respectively, 19, 12, and 17 days after administration (Fig. 5g). In marked contrast, the ALP values of dog F, and AST values of dogs D, E, and F in the POx-PSA group were within normal ranges (ALP, < 89 U/L; AST, 17–44 U/L). Dogs D and E were found to have slightly higher ALP (93–139 U/L) than the standard values, but no significant difference was found relative to the initial levels. The POx-PSA administration did not appear to affect hepatic function.

#### Inflammatory marker

When inflammation occurs in the body, C-reactive protein (CRP) is released into the blood. The CRP values of dogs A and B in the PSA group started to increase 9 days after sample infusion. They respectively reached maximum values of 21.7 and 23.6 mg/dL (Fig. 5h). The value of dog C also increased to 11.8 mg/dL at 17 days after administration. The CRP of dogs D and F in the POx-PSA group rose slightly to 4.6 mg/dL at 1 day and 14 days after administration, but they were much lower than the values of the PSA group.

## Discussion

### Synthesis and properties of POx-PSA

This report explained the synthesis and physicochemical properties of PSA wrapped covalently with poly(2-ethyl-2-oxazoline), POx-PSA, and described the demonstration of its safety and efficacy as an artificial plasma expander revealed using in vitro and in vivo experiments. The POx-PSA was prepared using a two-step coupling reaction between PSA and a unique POx-SH in high yield. Given the difficulty of introducing multiple processes to avoid infection and contamination, we chose PSA obtained from SPF porcine blood, which presents no risk of pathogens. When industrially produced, HSA is isolated from human donated plasma using the well-known Cohn method with cold ethanol fractionation^25^. However, this lengthy procedure requires specialized equipment. We have established a simple purification strategy of PSA using gentle heating alone. It can be conducted on both laboratory and industrial scales. Although many proteins denature around 60 °C, the albumin complexed with fatty acids showed high thermal stability at temperatures higher than 70 °C^26^. Excess unreacted POx was removed from the reaction mixture by TFF. Plasma expander is generally administered in large clinical doses. Our purification technique using TFF is beneficial for large-scale production. It is expected to be applicable to other combinations of therapeutic proteins and synthetic polymers.

The large particle size of POx-PSA compared to naked PSA suggests the several POx chains are coupled covalently to the core protein. Based on results on gravimetric analysis and MALDI-TOFMS, we concluded that the average number of POx linked to PSA is 6.0. The neutral surface net charge of POx-PSA also proves the enfolding of PSA having a negatively charged surface by the POx shell. The identical CD spectra of POx-PSA and PSA imply that the POx coating did not affect the secondary structure of the PSA core. The structures of the proteins are fundamentally the same in terms of their general features. The POx-PSA solution ([PSA] = 5 g/dL) showed sufficiently high COP (36 mmHg). For that reason, infusion of this hypertonic liquid after hemorrhagic shock is expected to raise the lowered circulating blood volume. This prediction was demonstrated to be true.

Lyophilization is an effective method to store proteins for long periods. Freezing and drying, however, cause denaturation and aggregation by the disruption of hydrogen bonds. To maintain the protein structure, sugars (e.g. sucrose and trehalose) are often used as lyoprotectants. Remarkably, lyophilized powder of POx-PSA can be stored for at least 1 year. It can be regenerated by the addition of deionized water, PBS, and saline solution. The POx decoration is likely to stabilize the PSA center during freeze-drying and thawing processes.

Upon addition of the POx-PSA solution ([PSA] = 5 g/dL) to whole blood, the RBC, WBC, and PLT counts decreased depending on the ratio of their addition (20–40 vol%); then they remained constant for 6 h at 37 °C. The findings indicate that POx-PSA is dispersed homogeneously in whole blood and that it did not induce change of blood cell counts. This stability signifies good compatibility of POx-PSA with the blood cell components. Blood dilution causes a decrease in the thrombin amount. Thereby, the blood coagulation time was prolonged slightly depending on the POx-PSA volume ratio. On the one hand, in the HES group, an increase of APTT was lower than that observed in the POx-PSA group. This may suggest blood coagulation disorder of HES. We conclude that POx-PSA is compatible with blood and that it does not influence coagulation systems.

### Preclinical studies of POx-PSA

The POx wrapping of PSA doubled the circulation lifetime of the protein in rats. We inferred that the superior blood retention property of POx-PSA can be attributed to its (i) moderately large molecular size and (ii) non-immunological feature. As expected, anti-PSA IgG antibody was not detected after administration of POx-PSA to rats. The POx decoration confers excellent immunological stealth properties to PSA. It is noteworthy that no anti-POx IgG antibody was generated either. These results contrast sharply against the fact that the injection of PEG-PSA engendered the production of anti-PSA IgG and anti-PEG IgG antibodies. We reasoned that the PEG coating was insufficient to block the immunogenicity of PSA in our preparation. It became apparent that conjugation of POx chains to PSA enables us to mask its antigenicity. The resulting POx-PSA was found to be an immunologically free preparation.

Hemorrhagic shocked rats were resuscitated efficiently by administration of the POx-PSA solution, as revealed by improvements of circulatory parameters and blood gas parameters. The restored MAP and HR values remained constant for 2 h, demonstrating maintenance of the circulating blood volume. Results showed clearly that administration of the POx-PSA solution is beneficial for recovery from hypotension and bradycardia by hemorrhagic shock. The resuscitation effect of the POx-PSA group was superior to that observed for the control HES group, which had been infused with the Voluven solution. This difference can be ascribed to the longer circulation lifetime of POx-PSA. Approximately 80% of POx-PSA remains in the bloodstream 2 h after injection. Although the HES solution possesses high COP (35 mmHg), which is comparable to that of POx-PSA, the synthetic polymer has been excreted quickly. One possible explanation is that clearance of HES from the bloodstream would reduce oncotic pressure and diminish the circulating blood volume. For that reason, blood pressure and HR, after once recovering, fell within 2 h. In the HES group, the RBC counts increased slightly after 1 h. This rise is also interpreted as a reduction of circulating blood volume. The pH and lactate values reverted to the basal values by 0.5 h after injection of POx-PSA and remained constant for 2 h. The acid–base equilibrium must have returned to normal levels. These results signify that a moderate amount of O_2_ was supplied to peripheral tissues by adequate circulating blood volume. The results of serum biochemistry tests indicated that the administration of POx-PSA caused no disorder in liver or kidney function. The minor histopathological changes observed in lungs, liver, and kidneys resulted from hemodynamic abnormalities arose from blood bleeding and reperfusion. They were not related to the administered substances. Based on all these findings, we concluded that the POx-PSA solution is a superior resuscitative fluid for hemorrhagic shock to achieve (i) restoration of circulatory volume compared to the HES solution, which is widely used in veterinary medicine, and (ii) resolution of abnormalities in circulation, hematological, and blood gas parameters without burdening the liver and kidneys.

An important finding is that the administration of the POx-PSA solution to healthy dogs caused no side-effect, adverse event, or severe inflammatory reaction, attesting to the adequate safety of the formulation. This finding rather contrasts to the fact that severe adverse events were observed in the PSA group. Actually, PSA is a heterologous protein for dogs. Its clinical use without surface decoration as a plasma expander presents great risk. Indeed, infusion of PSA in dogs induced abnormalities in renal and hepatic parameters, indicating not only late acute kidney injury, but also liver dysfunction. The considerably higher CRP values of the PSA group suggest that the adverse events appear to be attributable to delayed allergic reactions. By contrast, the CRP values in the POx-PSA group were much lower than those of the PSA group, suggesting that no adverse event occurred because of an allergic reaction. Overall, POx-PSA possesses three important beneficial features: (i) low immunogenicity, (ii) long-term circulation, and (iii) adequate in vivo safety for clinical use. The POx-PSA solution can be of great medical importance as a potential artificial plasma expander used in diverse veterinary medicine situations.

## Limitations of the study

For this study, after synthesizing a POx-PSA preparation designed for use as a new artificial plasma expander for dogs, we clarified its safety and efficacy using animal experiments. Although its safety was demonstrated by administration to dogs, testing was limited to a small number (total six) of healthy dogs because of veterinary ethical issues. In future studies, it will be important to conduct efficacy evaluations using disease models that closely resemble clinical conditions. Moreover, the in vivo metabolism and excretion of POx-PSA have not yet been elucidated.

## Methodology

### Material and apparatus

Fresh porcine blood from SPF swine was purchased from Tokyo Shibaura Zouki Co., Ltd. Poly(2-ethyl-2-oxazoline) (POx) (Mw: 5 kDa) and 4,4′-dithiodipyridine (4DTP) were purchased from Merck KGaA. 3,3′-Dithiodipropionic acid (DTDPA), 4-dimethylaminopyridine (DMAP), *N*,*N*′-dicyclohexylcarbodiimide (DCC), and *N*-succinimidyl 3-maleimidopropionate (SMP) were purchased from Tokyo Chemical Industry Co., Ltd. (+/−)-Dithiothreitol (DTT) was purchased from Fujifilm Wako Pure Chemical Corp. A fluorescence labeling agent, Cyanine5.5(Cy5.5)NHS ester, was purchased from Lumiprobe Corp. A hydroxyethyl starch (HES) solution (Voluven 6% solution for infusion) was purchased from Otsuka Pharmaceutical Co., Ltd. POx-1,2-distearoyl-sn-glycero-3-phosphoethanolamine (POx-DSPE) was purchased from Ruixibiotech Co. Ltd. PEG-1,2-dipalmitoyl-sn-glycero-3-phosphoethanolamine (PEG-DPPE) was purchased from NOF Corp. All other chemical reagents were purchased from commercial sources as special grades and were used without further purification. Deionized water (18.2 Mcm) was prepared using a water purification system (Milli-Q IQ7003 system; Merck KGaA).

### Synthesis of POx-SH

To a dehydrated THF solution (60 mL) of POx (5 g, 1.0 mmol), DTDPA (1.26 g, 6.0 mmol), and DMAP (0.16 g, 1.3 mmol) in a round-bottom flask (200 mL volume), DCC (1.36 g, 6.6 mmol) was added. Then the mixture was stirred for 18 h at 25 °C. After the solvent was removed using a rotary evaporator under reduced pressure, the white residue was dried *in vacuo*. The powder was redissolved in deionized water (20 mL) and the precipitate was removed by centrifugation (14,000×*g*, 30 min). After adjusting the solution pH to 8.0 using 0.1 M aqueous NaOH, DTT (1.39 g, 9.0 mmol) was added. The resultant mixture was stirred for 2 h at 25 °C under N_2_. The obtained solution was dialyzed (MWCO, 3.5 kDa) against deionized water at 4 °C and was freeze dried under reduced pressure, yielding white powder of POx having a terminal sulfhydryl group (POx-SH). The formation of POx-SH was revealed by sulfhydryl group assay using 4DTP and ^1^H NMR spectroscopy. The sulfhydryl group ratio was 100%. The yield was 80%.

### Purification of PSA from porcine blood

Fresh blood of SPF swine (4 L) was centrifuged (1000×*g*, 30 min, 4 °C). The supernatant (plasma) was frozen at − 80 °C. After this fresh-frozen plasma (FFP) was thawed slowly at 4 °C, the cryoprecipitate was removed by centrifugation (4000×*g*, 30 min, 4 °C) to obtain porcine serum ([PSA] = 2.8 g/dL). Sodium caprylate solution (0.3 M) was added to the serum (30 μmol/mL-plasma) and pH was adjusted to 6.8 by dropping 5 M HCl. The half of this solution was transferred into a round-bottom flask (2 L volume) and was heated at 70 °C for 1 h with vigorous stirring. After cooling the solution, pH was adjusted to pH 4.5 by the addition of 5 M HCl. The obtained dispersion was centrifuged (10,000×*g*, 30 min, 4 °C) to remove denatured proteins. After the supernatant was diluted three times with deionized water, the pH was adjusted to 8.0 using 5 M NaOH. The same heat treatment was performed on the remaining half amount of the serum added sodium caprylate. Then all resulting solution was subjected to anion exchange chromatography (AEC) with Q Sepharose Big Beads (800 mL; Cytiva) using Tris–HCl solution (50 mM, pH 8.0) as a running buffer. After flushing the Tris–HCl buffer (50 mM, pH 8.0, + 40 mM NaCl) to wash out unnecessary proteins, PSA was eluted using sodium phosphate (PB) buffer (10 mM, pH 7.4, + 187 mM NaCl). The eluent was dialyzed (MWCO, 12‒14 kDa) against deionized water at 4 °C and was condensed using an ultrafilter (P0200, MWCO, 20 kDa; Advantech Co., Ltd.) in an ultraholder (UHP-76K; Advantech Co., Ltd.) at 25 °C. The obtained PSA was analyzed using SDS-PAGE (SuperSep Ace 5–20%; Fujifilm Wako Pure Chemical Corp.) and size exclusion chromatography (SEC) on an HPLC system (Prominence LC-20 AD/CTO-20A/SPD-20A; Shimadzu Corp.) with an SEC column (YMC-Pack Diol-300 S-5; YMC Co. Ltd.) using 50 mM PB pH 7.4) as a mobile phase.

### Synthesis of POx-PSA

Freshly prepared DMSO solution of SMP (150 mM, 8 mL) was added dropwise into PBS solution of PSA (1 mM, 80 mL) in a round-bottomed flask (300 mL volume). The mixture was stirred for 1 h at 25 °C. After removing unreacted excess SMP using a GFC column (Sephadex G-25 superfine; Cytiva) with PBS as a running buffer, the volume of the obtained maleimide-activated PSA (MA-PSA) solution was adjusted to 240 mL ([PSA] = 0.33 mM) by adding PBS. Then, after PBS solution of POx-SH (3.9 mM, 160 mL) was added slowly to the MA-PSA solution, the mixture was stirred for 24 h at 25 °C. The resultant (total 400 mL) was dialyzed against deionized water at 25 °C to remove unreacted excess POx-SH using a tangential flow filtration (TFF) system (Pellicon XL Ultrafiltration Module Biomax 100 kDa; Merck KGaA), with subsequent addition of 5.3% volume of 20 × PBS. Purity of POx-PSA was measured using SEC on an HPLC system (Extrema PU-4180/UV-4075; Jasco Corp.) with a SEC column (Superdex 200 Increase; Cytiva) using PBS as mobile phase.

Dynamic light scattering (DLS) measurements of POx-PSA ([PSA] = 10 μM, in PBS) were conducted using a zeta-potential and particle size analyzer (ELSZ-2000ZS; Otsuka Electronics Co., Ltd.) at 25 °C. The hydrodynamic diameters were estimated using numbers from the particle size distribution. The zeta potential of POx-PSA ([PSA] = 20 μM, in PBS) was also measured using ELSZ-2000ZS at 25 °C.

The average number of POx chains bound to POx-PSA (*m*) was ascertained from results of (i) gravimetric analyses of freeze-dried powder and (ii) mass spectrometry. (i) An aqueous solution of POx-PSA ([PSA] = 0.5 mM, 0.7 mL) was frozen in liquid N_2_ bath for 5 min. It was subsequently freeze-dried under reduced pressure for 18 h using a freeze dryer (FDU-1200; Tokyo Rikakikai Co., Ltd.). The *m* value was calculated from the mass of the lyophilized powder. (ii) Mass spectra of POx-PSA were obtained using MALDI-TOF mass spectroscopy (MALDI-8020; Shimadzu Corp.) (linear flight mode, positive ion detection mode). The specimens were prepared by mixing a sample solution (1–2 μL) with matrix solution (20 mg/mL sinapinic acid in 50% aqueous CH_3_CN, 0.5 μL) on the measuring plate and air-drying with subsequent on-plate desalting with 0.1% TFA, addition of matrix (0.5 μL), and air-drying. The peak pattern simulation and curve fitting were performed using AXIMA series (Shimadzu Corp.) software. From the fitting spectral results, each existence ratio of POx-PSA with different PSA number was determined. The *m* value was calculated.

PEG-PSA was prepared via the same procedure using poly(ethylene glycol) with a sulfhydryl terminus (Mw, 5 kDa, Sunbright ME-050SH; NOF Corp.).

### CD spectrum measurement and COP measurement

Circulation dichroism (CD) spectrum of POx-PSA ([PSA] = 4 μM, in PBS) was measured using a CD spectrophotometer (J-1100; Jasco Corp.) at 25 °C.

Colloid osmotic pressure (COP) of POx-PSA ([PSA] = 1, 2, 3, 4, and 5 g/dL, in PBS) were measured using an automatic colloid-osmometer (OSMOMAT 050; Gonotec GmbH) with a membrane (MWCO, 20 kDa) at 25 °C.

### Blood cell counts

Fresh whole blood was obtained from Wistar rats (approximately 232 g, male) and stored in EDTA-2Na coated blood collection tubes. The POx-PSA solution ([PSA] = 5 g/dL, in PBS) was added to whole blood at 0, 10, 20, and 40 vol% concentrations (total volume 0.6 mL each). Individual samples were incubated at 37 °C in an incubator. At the time-points of 0, 1, 2, 3, 4, 5, and 6 h after the mixing, a sample (50 μL) was drawn from each tube. The quantities of blood cell components (RBC, WBC, and PLT) were assessed using an automated hematology analyzer for animals (pocH-100iV Diff; Sysmex Corp.) (*n* = 3). The results are shown as a percentage: (cell number with POx-PSA)/[cell number without POx-PSA (basal value at each time-point)].

### Prothrombin time (PT) and activated partial thromboplastin time (APTT)

To the citrated whole blood of rat, the POx-PSA ([PSA] = 5 g/dL, in PBS) was added at 0, 10, 20, and 40 vol% concentration (total volume 1.2 mL each) in different blood collection tubes (B-11; BML Inc.). After centrifugation (2800 rpm, 15 min), the supernatant was transferred to blood collection tube (S-1; BML Inc.) and was frozen at − 80 °C. The whole blood mixed with HES (Voluven) solution instead of POx-PSA was used as a control. The PT and APTT measurements were performed by BML Inc. (Tokyo) (*n* = 3).

### Blood circulation persistence in rats

Fluorescence probe (Cy5.5) labeled POx-PSA [POx-PSA(Cy5.5)] was synthesized as follows. The DMSO solution of Cy5.5 NHS ester (18 mM, 58 μL) was added dropwise into the POx-PSA solution (0.6 mL, [PSA] = 0.9 mM, in 0.1 M PB, pH 8.4) ([Cy5.5 NHS ester]/[PSA] = 2 mol/mol). Then the mixture was stirred for 18 h at 4 °C in the dark. The resultant was subjected to GFC (Sephadex G-25 superfine; Cytiva) to remove the unreacted Cy5.5 NHS ester with PBS as a running buffer. The [Cy5.5]/[PSA] ratio of POx-PSA(Cy5.5) obtained from UV–vis and CD spectral measurements was 1.1. Unlabeled POx-PSA ([PSA] = 5 g/dL) and POx-PSA(Cy5.5) were mixed to prepare a formulation ([Cy5.5]/[PSA] = 0.1, mol/mol).

Wistar rats (male; 233 ± 6 g weight; *n* = 4) were placed on a thermal pad (DC temperature controller; Brain Science Idea, Co., Ltd.) under sevoflurane (4.0% in air; Maruishi Pharmaceutical Co., Ltd.) inhalation anesthesia. A catheter (0.5 mm inner diameter, 0.8 mm outer diameter, SP-31; Natsume Seisakusho Co., Ltd.) was inserted into the right jugular vein. The opposite tip was fixed subcutaneously to the posterior neck. POx-PSA(Cy5.5) solution was administered to anesthetized rats from the right jugular vein (140 mg/kg-rat). After blood (200 μL) was collected from the right jugular vein at 0, 5, 15, and 30 min, and at 1, 3, 6, 12, 18, and 24 h after administration, it was centrifuged (6000 rpm, 5 min) to obtain serum (100 μL). Aqueous Triton X-100 solution (1.5 w/v%, 40 μL) was added to each serum sample (20 μL). Then the mixture was incubated for 16 h at 4 °C in the dark. The fluorescence spectrum of POx-PSA(Cy5.5) in this solution was measured using a fluorescence spectrophotometer (λ_ex_ = 684 nm, λ_em_ = 710 nm, FP-8300; Jasco Corp.). The elimination half-lives (t_1/2_) in blood in the β phase, AUC, MRT, CL_tot_ and V_dss_ of POx-PSA were calculated from the decay curve of the fluorescence intensity using a two-compartment model. As a reference group, PSA(Cy5.5) solution was also administered.

### Immunogenicity test of rats (2-dose test)

POx-PSA solution (200 mg/kg-rat) was injected to Wistar rats (male; 179 ± 9 g weight; *n* = 3) from the tail vein under sevoflurane inhalation anesthesia. Blood (100 μL) was collected from the tail vein before (0 day) and at 7, 14, 21, and 28 days after administration. Moreover, the same volume of POx-PSA solution was administered 28 days after injection. In addition, 100 μL of blood was collected from the tail vein at 35, 42, 49, and 56 days after injection. The serum component (50 μL) was separated by centrifugation and was stored at − 80 °C. As reference groups, PSA and PEG-PSA solutions were administered (*n* = 3 each).

Anti-PSA IgG antibody in each serum was assayed using ELISA. Each well in a 96-well plate was coated with PSA (100 μg/mL, in PBS, 100 μL) and was washed three times with wash solution (200 μL, 50 mM Tris–HCl, + 0.14 M NaCl, + 0.05% CHAPS). Then, blocking buffer (200 μL, 50 mM Tris–HCl, + 0.14 M NaCl, + 2% skim milk) was added, followed by incubation for 1 h at 25 °C. After washing three times, a serum sample (100 μL) diluted 50 times with blocking buffer was added. After incubation for 1 h at 25 °C, the plate was washed three times. HRP-labeled IgG (Rat IgG-Fc Fragment Antibody) (200 μL) diluted 1000-times with blocking buffer was added. After incubation for 1 h at 25 °C, the plate was washed three times. Subsequently, the *o*-phenylenediamine (OPD) solution [OPD tablet (1 tablet/10 mL), H_2_O_2_, phosphate-citrate (100 mM, pH 5.0)] was added to each well (95 μL/well). Then the sample was incubated for 10 min at 25 °C. The reaction was stopped by stop solution (1 M H_2_SO_4_, 100 μL/well). Then absorbance (490 nm) of each sample was measured using a microplate reader (Multiskan SkyHigh; Thermo Fisher Scientific Inc.).

Anti-POx IgG antibody in each serum in the POx-PSA group was assayed using the same method using a 96-well plate coated with POx-1,2-distearoyl-sn-glycero-3- phosphoethanolamine (POx-DSPE, 50 μM, in H_2_O, 100 μL). Anti-PEG IgG antibody in the PEG-PSA group was assayed using PEG-1,2-dipalmitoyl-sn-glycero-3-phosphoethanolamine (PEG-DPPE).

### Resuscitation from hemorrhagic shock rats

**Figure 6:**
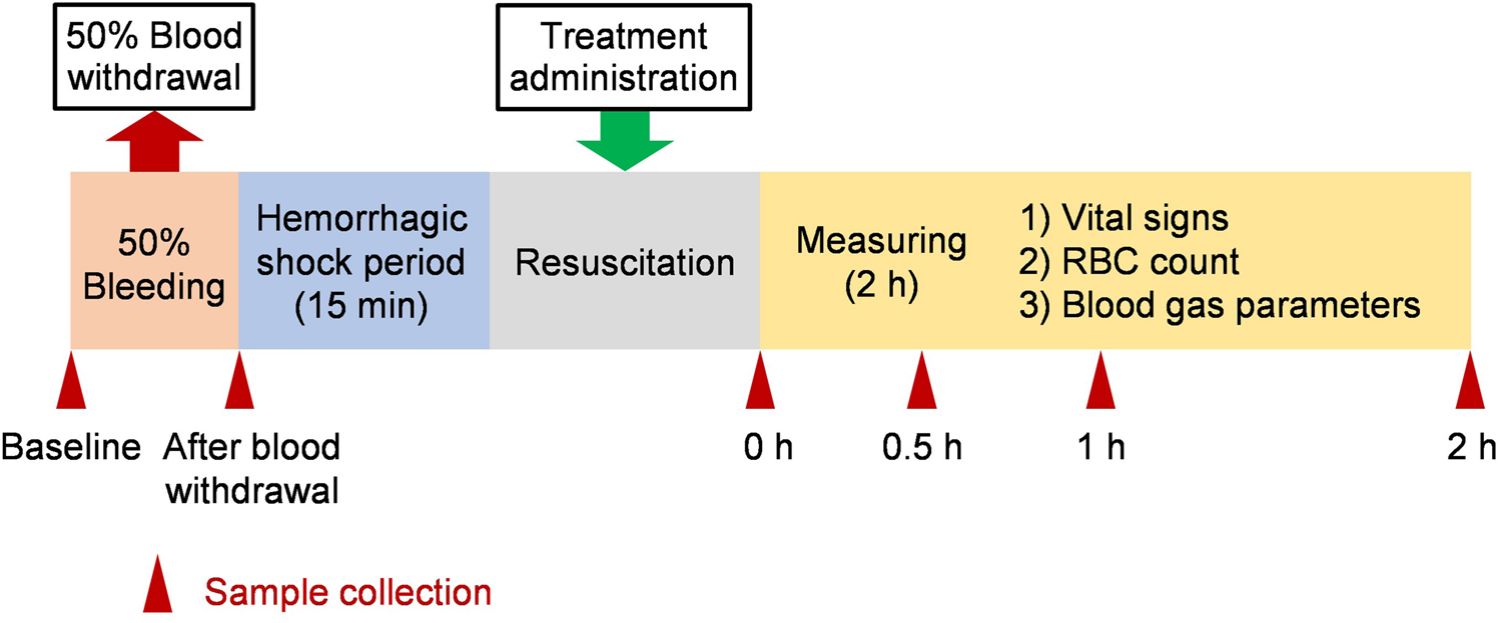
Timeline showing 2 h observations after resuscitation from hemorrhagic shock in a rat model.

Wistar rats (male; 222 ± 11 g weight; *n* = 6) were placed on a thermal pad (DC temperature controller; Brain Science Idea Co., Ltd.) under sevoflurane (4.0% in air; Maruishi Pharmaceutical Co., Ltd.) inhalation anesthesia. The anesthesia and temperature control were maintained through all experiments (2 h after administration). The animals were breathing spontaneously. A catheter (SP-31) was inserted into the right carotid artery for continuous monitoring of the MAP using a blood pressure measurement device (PAS-101; Star Medical, Inc.) and for blood withdrawal. A different catheter was inserted into the right jugular vein for administration. The rat was tracheotomized and ventilation was controlled using a ventilator (Small Animal Ventilator 683; Harvard Apparatus) to keep the *P*aO_2_ value of approximately 100 mmHg [sevoflurane (3.0% in 30 ± 3% O_2_), 11 ± 1 mL/kg ventilation volume per cycle, 82 ± 1/min respiratory rate]. A timeline of experiments is presented as Fig. 6. After stabilization of the animal condition (after approximately 30 min), hemorrhagic shock was induced by bleeding 50% of the total blood volume from the carotid artery (1 mL/min). After 15 min, the 60% of the bleeding volume (30% of the initial blood volume) of POx-PSA solution ([PSA] = 5 g/dL, in PBS) (*n* = 6) was administered from the jugular vein for resuscitation (1 mL/min). The circulation parameters, especially vital signs (MAP and HR) were measured at the following ten time points: before 50 vol% blood withdrawal, immediately after 50 vol% blood withdrawal, immediately before administration and at 0 (immediately after administration), 5, 15, and 30 min, and 1, 1.5, and 2 h after administration. Arterial blood (0.2 mL) was collected for hematological and blood gas analyses at the following six time points: before 50 vol% blood withdrawal, immediately after 50 vol% blood withdrawal, and at 0 (immediately after administration) and 30 min, and 1 and 2 h after administration. Blood samples were collected using anticoagulant-free syringe and immediately subjected to the measurements. The RBC count was found using a multi-item automatic hematology analyzer (pocH-100iv Diff; Sysmex Corp.). The *P*aO_2_,* P*aCO_2_, pH, and the lactate value were measured using a portable blood gas analyzer (i-STAT; Abbott Point of Care Inc.) with a test cartridge (i-STAT test cartridge CG4+; Abbott Point of Care Inc.). As a reference group, shocked rats were resuscitated by administering HES (Voluven) solution (HES group, *n* = 6). At 2 h after administration, arterial blood (5 mL) was withdrawn from the carotid artery and was transferred into a vacuum blood collection tube containing a serum-separating agent (VenojectII, VPP075K; Terumo Corp.). After centrifugation (2580×*g*, 10 min), the serum (1.5 mL) was stored at − 80 °C. All samples were subjected to serum biochemical tests by BML Inc. (Tokyo, Japan): albumin, A/G, AST, ALT, LDH, BUN, Cre, and K^+^. The blood withdrawn for inducing hemorrhagic shock at first was also measured; the data were treated as baselines (basal values) for the respective items. After the experiments, the animals were euthanized using a ketamine/xylazine overdose. Organs (heart, lung, liver, kidney, and spleen) were fixed in 20% formalin solution. Paraffin sections were stained with hematoxylin/eosin.

### Safety evaluation of dogs (top load)

Healthy adult beagle dogs (castrated males A, B, D, and E, spayed females C and F; 10.0 ± 1.2 kg weight; 2–8 years old; *n* = 6) were used for this study. Removal of contaminating lipopolysaccharides in the formulation was performed by Nagase ChemteX Corp. Saline solution of POx-PSA ([PSA] = 5 g/dL) was administered intravenously from the cephalic vein using a syringe pump during 30 min under awake conditions (5 mL/kg-dog) (dogs D, E, and F). As a reference group, saline solution of PSA ([PSA] = 5 g/dL) was administered by the same procedure (5 mL/kg-dog) (dogs A, B, and C). General conditions of the beagles were observed for 28 days after administration. Venous blood (1.0 mL) was collected at the following 17 time points: at 0 (before administration), 1, 2, 3, 4, 5, 6, 7, 8, 9, 10, 11, 12, 13, 14, 21, and 28 days after administration. The obtained blood was subjected to complete blood counts and blood biochemical examinations (albumin, BUN, Cre, ALP, AST, and CRP). Additional blood tests and treatment with medications were conducted as needed based on results of evaluations by veterinarians (Table 2). The additional data were also shown in Fig. 5.

### Statistical analysis

Data of in vivo experiments are expressed as mean ± SD for the designated number of animals. Differences for each parameter between the groups were analyzed using two-tailed unpaired Student’s *t* tests assuming normal distribution. Analyses were performed using Microsoft Excel software (Microsoft Corp.). Results for which the *P* value was < 0.05 were inferred as statistically significant.

### Approval for animal experiments

All animal experiments were carried out in accordance with ARRIVE guidelines^27^ and approved by the Animal Care and Use Committee of Tokai University, Keio University, and The University of Tokyo. The care and handling of the animals were performed in accordance with NIH guidelines.

## Supplementary Information

Supplementary Figure S1.

## Data Availability

The datasets generated during and/or analyzed during the current study are available from the corresponding author (T. Komatsu) on reasonable request.
