# Supplementary material for: Polyoxazoline-conjugated porcine serum albumin as an artificial plasma expander for dogs
Source: Sci Rep. 2023 Jun 14;13:9512. doi: 10.1038/s41598-023-35999-4 (PMC10267181; doi:10.1038/s41598-023-35999-4)
Supplement: Supplementary file 1 — Supplementary Figure S1. [file 41598_2023_35999_MOESM1_ESM.pdf]

Supplementary Information

Polyoxazoline-conjugated porcine serum albumin as an artificial plasma expander for dogs

Wataru Okamoto[1], Tomone Usui[1], Mai Hasegawa[1], Tatsuhiro Kobayashi[1], Junya Fujisawa[1], Kazuaki Taguchi[2], Kazuaki Matsumoto[2], Mitsutomo Kohno[3], Masayuki Iwazaki[4], Shotaro Shimano[5], Itsuma Nagao[5], Hiroto Toyoda[5], Naoki Matsumura[5], Hirotaka Tomiyasu[5], Ryota Tochinai[6] & Teruyuki Komatsu[1,]*

[1] *Department of Applied Chemistry, Faculty of Science and Engineering, Chuo University, 1-13-27 Kasuga, Bunkyo-ku, Tokyo 112-8551, Japan*

[2] *Division of Pharmacodynamics, Faculty of Pharmacy, Keio University, 1-5-30 Shibakoen, Minato-ku, Tokyo 105-8512, Japan*

[3] *Department of General Thoracic Surgery, Saitama Medical Center, Saitama Medical University, 1981 Kamoda, Kawagoe-shi, Saitama 350-8550, Japan*

[4] *Department of Thoracic Surgery, School of Medicine, Tokai University, 143 Shimokasuya, Isehara-shi, Kanagawa 259-1193, Japan*

[5] *Department of Veterinary Internal Medicine, Graduate School of Agriculture and Life Sciences, The University of Tokyo, 1-1-1 Yayoi, Bunkyo-ku, Tokyo 113-8657, Japan*

[6] *Department of Veterinary Pathophysiology and Animal Health, Graduate School of Agriculture and Life Sciences, The University of Tokyo, 1-1-1 Yayoi, Bunkyo-ku, Tokyo 113-8657, Japan*

*Corresponding author: Prof. Teruyuki Komatsu

Tel & Fax: +81-3-3817-1910, E-mail: komatsu@kc.chuo-u.ac.jp

Result

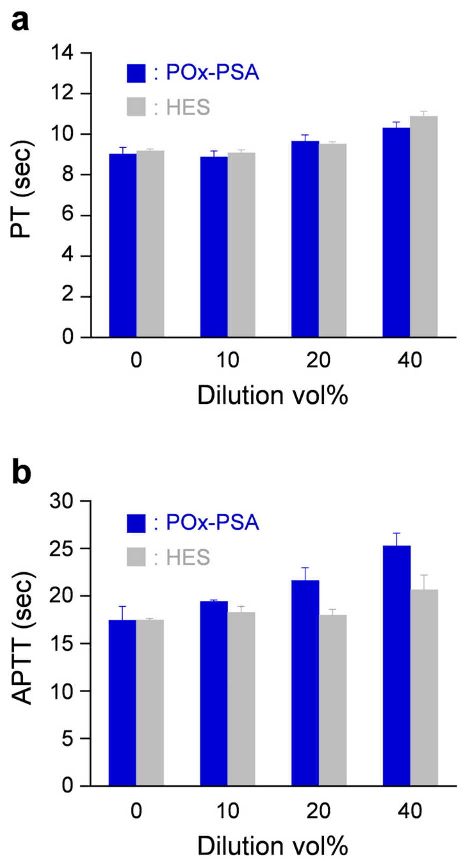

Figure S1. (a) PT and (b) APTT values of blood samples after mixing with POx-PSA. Each datum represents mean ± SD (n = 3).
